# Imaging the structural connectome with hybrid MRI-microscopy tractography

**DOI:** 10.1016/j.media.2025.103498

**Published:** 2025-02-06

**Authors:** Silei Zhu, Istvan N. Huszar, Michiel Cottaar, Greg Daubney, Nicole Eichert, Taylor Hanayik, Alexandre A. Khrapitchev, Rogier B. Mars, Jeroen Mollink, Jerome Sallet, Connor Scott, Adele Smart, Saad Jbabdi, Karla L. Miller, Amy F.D. Howard

**Affiliations:** a Wellcome Centre for Integrative Neuroimaging, FMRIB Centre, Nuffield Department of Clinical Neurosciences, University of Oxford, Oxford, United Kingdom; b Wellcome Centre for Integrative Neuroimaging, Experimental Psychology, Medical Sciences Division, University of Oxford, Oxford, United Kingdom; c Department of Oncology, University of Oxford, Oxford, United Kingdom; d Donders Institute for Brain, Cognition and Behaviour, Radboud University Nijmegen, Nijmegen, the Netherlands; e INSERM U1208, Stem Cell and Brain Research Institute, University Lyon, Bron, France; f Division of Clinical Neurology, Nuffield Department of Clinical Neurosciences, University of Oxford, Oxford, United Kingdom; g Department of Bioengineering, Imperial College London, London, United Kingdom

**Keywords:** Diffusion MRI, Microscopy, Brain connectivity, White matter fibre, Tractography

## Abstract

Mapping how neurons are structurally wired into whole-brain networks can be challenging, particularly in larger brains where 3D microscopy is not available. Multi-modal datasets combining MRI and microscopy provide a solution, where high resolution but 2D microscopy can be complemented by whole-brain but lowresolution MRI. However, there lacks unified approaches to integrate and jointly analyse these multi-modal data in an insightful way. To address this gap, we introduce a data-fusion method for hybrid MRI-microscopy fibre orientation and connectome reconstruction. Specifically, we complement precise “in-plane” orientations from microscopy with “through-plane” information from MRI to construct 3D hybrid fibre orientations at resolutions far exceeding that of MRI whilst preserving microscopy’s myelin specificity, resulting in superior fibre tracking. Our method is openly available, can be deployed on standard 2D microscopy, including different microscopy contrasts, and is species agnostic, facilitating neuroanatomical investigation in both animal models and human brains.

## 1 Introduction

Mapping brain structure through multiple modalities can improve our understanding of brain function, development, aging and diseases (Hansen et al., 2022; Smith et al., 2020; Sporns, 2013). Much effort has gone into creating comprehensive maps of brain connectivity using various methods from MRI to microscopy (Essen et al., 2013; Oh et al., 2014; Scheffer et al., 2020; White et al., 1986). However, mapping the whole-brain networks in larger brain is challenging. Different modalities can provide complementary information about the underlying tissue microstructure by probing different length scales and providing sensitivity to different tissue features. Notably, when multiple modalities are combined in the same tissue, this can offer new opportunities for multi-scale neuroscience.

Chemical tracers have been an important tool for mapping the pathways of neural connections in animal brains over many years (Jbabdi et al., 2015; Lein et al., 2007; Maffei et al., 2022; Markov et al., 2012). The tracer facilitates direct visualisation of axonal projection from an injection site, providing a highly specific and sensitive estimate of inter-regional brain connectivity (Markov et al., 2012, 2010). However, tracers are limited to a single or a few injections per animal, where achieving whole-brain coverage requires sacrificing many animals, and combining information across subjects ignores important between-subject variability (Lein et al., 2007; Markov et al., 2012, 2010). Further, tracers are prohibitive to use in humans.

Alternative microscopy techniques serve as crucial reference measurements for characterising fibre architecture at high spatial resolution (Mollink et al., 2017). For microscopy to be useful in tract reconstruction, the microscopy must inform on the fibre orientations in 3D. Multiple microscopy methods can provide 3D information (e.g. 3D polarised light imaging [3D-PLI] (Axer et al., 2011; Schubert et al., 2017; Reckfort et al., 2015), μ-CT (Dyer et al., 2017; Masís et al., 2018), SAXS (Georgiadis et al., 2020) or serial EM (Denk and Horstmann, 2004)), and synaptic organisation at the cubic-millimetre level has been mapped in the human cortex (Shapson-Coe et al., 2024). However, their sophisticated hardware and long acquisition times limit widespread application. Consequently, microscopy orientations are often acquired in 3D from only small tissue samples or in 2D from thin sections of brain tissue, thus precluding 3D tract reconstruction or whole-brain connectivity estimates in primate brains.

In comparison, diffusion MRI (dMRI) enables non-invasive mapping of structural brain connectivity (Edgar and Griffiths, 2009). By measuring the diffusive motion of water molecules through the tissue, we can infer underlying fibre orientations and reconstruct white matter (WM) fibre bundles or tracts via tractography methods (Beaulieu, 2002). This facilitates whole-brain estimation of white matter organisation but relies on fibre orientations estimated via computational models from millimetre-scale MR signals. Low resolution and inaccuracies in the models can introduce bias or noise in the inferred fibre orientations that contribute to false positives and negatives in downstream tractography outputs (Maier-Hein et al., 2017; Nath et al., 2020; Schilling et al., 2017; Thomas et al., 2014). Regardless, due to the substantial promise of the method for non-invasive brain connectivity imaging, extensive efforts have gone into both understanding and overcoming these tractography limitations, through validation studies and methodological advancements (Ambrosen et al., 2020; Aydogan and Shi, 2018; Caminiti et al., 2021; Côté et al., 2013; Delettre et al., 2019; Dell’Acqua, 2024; Donahue et al., 2016; Dyrby et al., 2007; Girard et al., 2020; Maffei et al., 2022; Schilling et al., 2019a, 2019c; Yendiki et al., 2022; Zhang et al., 2022).

**Fig. 1:**
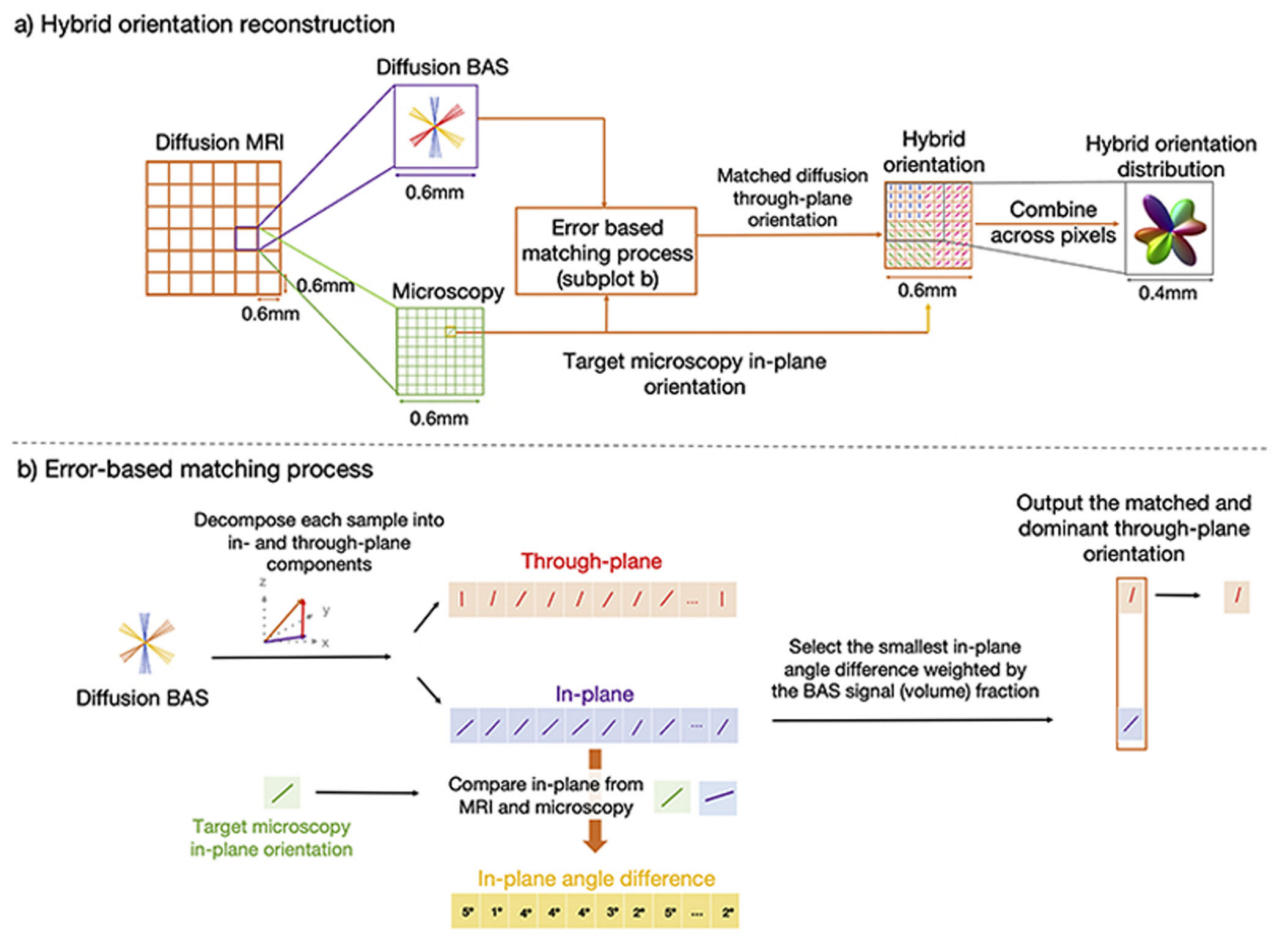
The hybrid MRI-microscopy approach. a) Our data fusion method leverages the complementary strengths of diffusion MRI and microscopy. Each 2D microscopy orientation was warped to dMRI space and compared to the BAS samples in the same voxel, where the BAS samples were first projected onto the microscopy plane to facilitate fair comparison with the 2D microscopy. The microscopy through-plane information was then approximated using that from the BAS sample selected by the error-based matching process. b) The error based matching process. The diffusion BAS and microscopy in-plane orientation were used as inputs. The 3D BAS orientation was decomposed into the through-plane angle and in-plane angle by projecting onto the microscopy plane. The in-plane angle was compared to the target microscopy in-plane orientation by quantifying the angle difference. We selected the BAS sample with the smallest in-plane angle difference weighted by the BAS signal fraction (related to the volume fraction where more dominant fibre bundles have higher signal fractions) and determined the matched through-plane angle.

Here, we propose a data-fusion approach (Howard et al., 2019) to jointly analyse data from MRI and microscopy to perform whole-brain microscopy-informed tractography. Our framework leverages the complementary information these modalities provide to create hybrid MRI-microscopy fibre orientations that are both 3D and at high resolution (Fig. 1), preserving the unique benefits of the microscopy whilst facilitating 3D tractography. We use 2D microscopy to provide detailed estimates of fibre orientation within the microscopy plane and MRI to provide the through-plane orientation. For the latter, we use models that estimate distributions of fibre orientations within MRI voxels (Behrens et al., 2003), rather than a single orientation per voxel (e.g. from diffusion tensor imaging) (Basser et al., 1994). By extracting the through-plane angle for the fibre within this distribution that best matches the in-plane orientation derived from microscopy, we can then estimate 3D fibre orientations at spatial resolutions that exceed the MRI data. The dMRI Ball and Stick (BAS) method produces many orientation samples per voxel. However, the precise sub-voxel spatial localisation of these fibre orientations is unknown. In our hybrid method, the microscopy essentially assigned the through-plane orientation of the 3D samples to their putative location within the voxel to “super-resolve” the MRI information. Our hybrid MRI-microscopy method provides: 1) 3D fibre orientations; 2) whole-brain coverage; 3) high resolution information and estimation of complex fibre architecture as described by the microscopy. The hybrid orientations are then combined into fibre orientation distributions (FODs) at arbitrary resolutions that are input into existing tractography pipelines for tract reconstruction. When based on myelin-sensitive microscopy, the hybrid orientations likely provide a more “myelin-specific” fibre orientation distribution than MRI, where the MRI FOD can represent a range of microstructural features including axons, dendrites and glia processes (Alexander et al., 2019; Jespersen et al., 2007; Stanisz et al., 1997). These myelin-specific FODs may be advantageous for certain applications such as defining the “myelin connectome” or tracking myelinated fibres into the cortex.

We demonstrate our approach using the BigMac dataset (Howard et al., 2023), an open-access multi-modal resource that includes postmortem dMRI and microscopy data from a single macaque brain with whole-brain coverage. The microscopy includes polarised light imaging (PLI) (Axer, 2011; Larsen et al., 2007; Schubert et al., 2017) as well as myelin- (Gallyas, 1971) and Nissl-stained histology (Kádár et al., 2009; Pilati et al., 2008). Precise co-registration between microscopy and MRI has been conducted, which is essential for meaningful data fusion at the voxel-wise level (Huszar et al., 2023a). Applying our hybrid method to the MRI and PLI data, we first demonstrate how to perform hybrid tractography at different resolutions and reconstruct microscopy-informed tracts spanning the whole macaque brain. With confidence in our method, we then demonstrate how hybrid tractography can be beneficial in neuroanatomical and methodological investigations. Specifically, we utilise our hybrid outputs to investigate two known challenges in MRI-based tractography: the gyral bias (Schilling et al., 2017) and bottleneck problem (Schilling et al., 2022), both of which are primarily driven by the limited spatial resolution of MRI data. By comparing our hybrid outputs to MRI results in the same brain, we investigate how these challenges are related to the resolution and contrast-generating mechanism of MRI. We then compare the hybrid tractography outputs to tracer data obtained from other animals (Markov et al., 2012, 2010) to demonstrate that our hybrid method provides higher specificity in fibre tracking than MRI only tractography. Finally, we perform hybrid tractography using three different microscopy contrasts (PLI, myelin- and Nissl-stained histology) to demonstrate its application on different types of microscopy. Overall, our method retains the benefits of microscopy without relying on invasive tracers, meaning we can estimate dense, microscopy-informed structural connectivity from a single brain, using a method that is translatable across species, including humans.

## 2 Methods

### 2.1 MRI and microscopy data acquisition

The BigMac dataset was previously acquired and pre-processed as described in Howard et al. (2023). Relevant to this work, an adult rhesus macaque brain was scanned postmortem on a 7T small animal scanner. Structural images were acquired with multi gradient echo sequence at a spatial resolution of 0.3 mm isotropic, FOV =76.8 × 76.8 × 76.8 mm, TE/TR = 7.8/97.7 ms, and flip angle = 30°.

Postmortem dMRI was acquired with spin echo 2D multi-slice sequence and single-line readout: 0.6 mm isotropic resolution data with 128 gradient directions at *b* = 4 ms/μm^2^ and 8 with negligible diffusion weighting, and 1 mm isotropic resolution data with 250 gradient directions at *b* = 4 ms/μm^2^ and 10 with negligible diffusion weighting.

After the scanning, the brain was sectioned into two blocks (anterior/posterior halves). Each block was sectioned into thin (50/100 μm) slices and allocated to one of six interleaved contrasts: polarised light imaging (PLI) (Axer, 2011; Axer et al., 2001, 2011; Larsen et al., 2007), Cresyl violet staining for Nissl bodies (Kádár et al., 2009; Pilati et al., 2008), Gallyas silver staining for myelin (Gallyas, 1971) and three unassigned sections that were stored for longevity. The slice thickness was 50 *μm* for 5 out of the 6 sections (including PLI, Nissl and myelin-stained sections) with one section 100 *μm* thick. Each section of the same contrast was repeated every 350 μm.

PLI estimated the primary fibre orientation based on the birefringence of myelinated axons with a resolution of 4 μm per pixel (Axer, 2011; Axer et al., 2001, 2011; Larsen et al., 2007). Images were acquired as the analyser (rotatable polariser) was rotated through 180°, with a 20° angular resolution. Images were background corrected after which a sinusoid was fitted to the measured intensity at each pixel (I) as a function of analyser rotation (ρ). The phase of the sinusoid described the in-plane fibre orientation. Retardance and transmittance maps were also calculated and here used only for visualisation and to drive the co-registration respectively.

Histology slides with Gallyas silver staining (myelin) and Cresyl violet staining (Nissl bodies) were digitised at a spatial resolution of 0.28 μm/pixel. 2D structure tensor analysis (Bigun et al., 2004; Budde and Annese, 2013; Budde and Frank, 2012) of the stained sections (using a Gaussian kernel with sigma=10 pixels) was used to estimate the fibre orientations for each microscopy pixel.

### 2.2 Constrained spherical deconvolution

The diffusion MRI data was analysed using constrained spherical deconvolution (CSD) (Tournier et al., 2007). CSD is a common fibre orientation estimation method that uses diffusion MRI data only and where the CSD-derived FODs and downstream tractography could be directly compared to equivalent outputs from the hybrid method (both FODs are similarly described using spherical harmonics). Here, CSD was performed using the dhollander algorithm for fibre response function estimation (Dhollander et al., 2016; Tournier et al., 2007).

### 2.3 MRI-Microscopy data co-registration

MRI-Microscopy registration was performed using TIRL (v3.1.1), an automated pipeline facilitating the accurate registration of the histology sections to the whole brain MRI via a sequence of linear and non-linear transformations (see (Howard et al., 2023; Huszar et al., 2023a, 2023b, 2022) for further details). Here each PLI, myelin- and Nissl-stained slide was registered independently to the MRI space. The structural MRI was used as a reference due to its high resolution and good grey/white contrast. Separately, the structural and dMRI were co-registered using linear registration in FSL (FLIRT) (Jenkinson et al., 2002; Jenkinson and Smith, 2001). By combining the TIRL and FLIRT transformations, the microscopy pixels were mapped to voxel coordinates in dMRI space. Mapping of the microscopy fibre orientations (vectors) to dMRI space also accounted for local rotations according to the warp field.

### 2.4 Hybrid MRI-microscopy fibre orientations

#### 2.4.1 Hybrid orientation reconstruction

To calculate the 3D hybrid MRI-microscopy orientations, microscopy provided the fibre orientations within the microscopy plane, and we approximated the through-plane angle (or inclination angle) with that from dMRI (BAS model). A schematic of the method is illustrated in Fig. 1.

##### 2.4.1.1 Estimating BAS orientations from diffusion MRI

The dMRI was analysed using the Ball and Stick (BAS) model (Behrens et al., 2003) to estimate ≤ 3 fibre populations with 50 orientation estimates (samples) per population. These sample orientations capture the uncertainty in the estimation of the 3 fibre populations, which in part reflects the distribution of fibre orientations throughout the dMRI voxel. Samples from BAS populations with signal fractions <0.05 were excluded from future analysis.

The BAS model was chosen due to its ability to estimate multiple fibre populations per voxel and provide discrete orientation samples that can be directly compared with those from microscopy. Other diffusion models providing estimation for the fibre orientation could also be used.

##### 2.4.1.2 Extracting 2D orientations from microscopy

PLI maps of fibre orientation within the microscopy plane (in-plane angles estimated for each pixel) were used as input for the hybrid MRI-PLI tractography. Inplane orientations were estimated from myelin- and Nissl- stained histology using 2D structure tensor analysis. No other processing was applied, and all in-plane orientations were considered equally throughout the tissue.

##### 2.4.1.3 Combing MRI and microscopy for hybrid orientation reconstruction

The microscopy in-plane orientations were first warped to dMRI space and compared with the co-registered BAS samples within the same voxel using an error-based matching process (Fig. 1b). To facilitate fair comparison of in-plane dMRI and microscopy orientations, the 3D BAS orientation samples were decomposed into the through-plane angle and in-plane angle by projecting the BAS orientation onto the microscopy plane. This process was repeated for ≤ 150 BAS samples per voxel (≤ 3fibre populations with 50 orientation samples per population). The in-plane angle of each sample was then compared to the target microscopy in-plane orientation by quantifying the angle difference (the “error-based matching process”, Fig. 1b).

In our initial studies, we then selected the hybrid through-plane angle as that from the BAS sample with the smallest in-plane angle difference to the microscopy. This through-plane diffusion orientation was then combined with the in-plane microscopy orientation to reconstruct the hybrid orientation which is both 3D and at microscopic resolution.

However, this matching process, based solely on the in-plane angular difference, was found to result in through-plane fibre populations (related to fibre bundles tracking along the anterior-posterior axis) being underestimated, with a bias for in-plane fibre populations (i.e. going left-right, or inferior-superior) which are better captured by the microscopy. Supplementary Figure 1 shows fibre orientations in the cingulum with a strong left-right orientation, though the cingulum primary bundle runs anterior-posterior. Consequently, the matching error was edited to be the combination of the angle difference and the signal fraction estimated from dMRI BAS (related to the volume fraction, where more dominant fibre bundles have higher signal fractions), to more accurately account for the relative contributions of different fibre populations. The probability, *p*_*i*_, of selecting BAS sample *i*, was calculated as the product of the BAS signal fraction and the cosine of the angular difference between the dMRI in-plane angle and the PLI angles. This approach assigned higher selection probabilities to samples with larger signal fractions and smaller angular differences between the BAS in-plane and PLI angles. We then selected our BAS sample using cumulative probability distribution sampling, a widely used machine learning method that is used to sample items from a list where each element has a specific weight or probability associated with it. The probabilities were first normalised to sum to one. The cumulative probabilities, *C*, were then computed by sequentially summing the individual probabilities for all elements (BAS samples), $C[i]=\Sigma_{j=1}^{i}p_{j}$. A random number, *r*, was then generated between 0 and 1 and we selected the BAS sample with the smallest index such that *C*[*i*] ≥ *r*. This approach ensured higher-probability elements were prioritised while maintaining randomness in the selection process. The selected BAS sample provided the through-plane angle for the hybrid orientation, as described above. With this approach, diffusion MRI has greater influence on the assignment of microscopy pixels to fibre populations, particularly when the in-plane angle is ambiguous (i.e. two distinct fibre populations have the same in-plane angle when projected onto the microscopy plane) and in regions where the fibre bundle runs primarily through the microscopy plane.

**Fig. 2:**
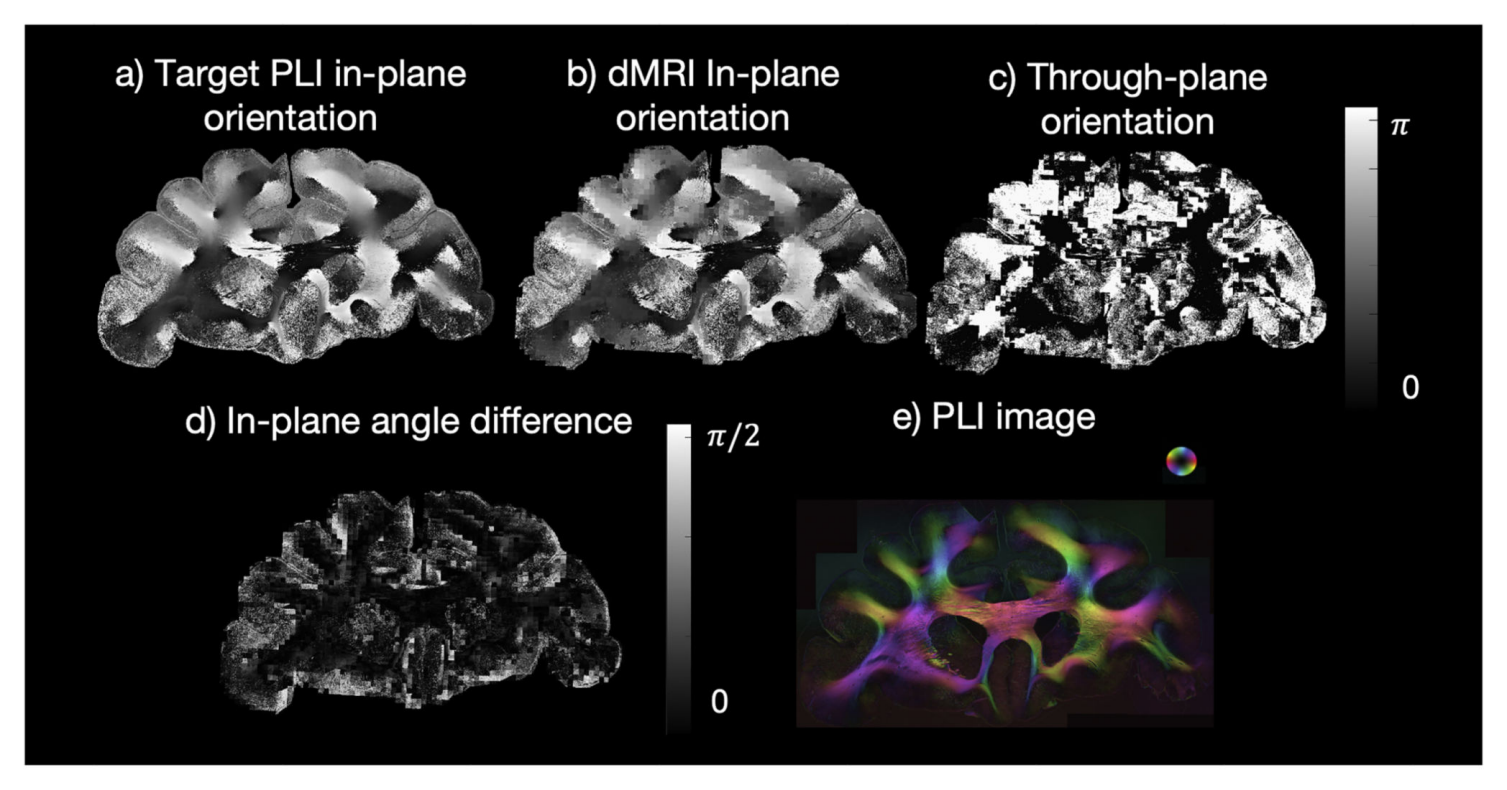
The angle component used in the hybrid orientation reconstruction. a) The PLI in-plane orientation. b) The in-plane orientation from the diffusion BAS decomposition (represented by the angle of the 3D dMRI vector projected onto the microscopy plane). Here we show the in-plane angle of the BAS sample that is most similar to the PLI. c) The through-plane orientation from the diffusion BAS decomposition (inclination angle); d) The in-plane angle difference between the target PLI orientation and the most similar BAS fibre orientation showing a small angle difference. e) The PLI hue-saturation-value with the colour-coded orientations.

Fig. 2 shows the in-plane, through-plane angle and angle difference maps for matched PLI and dMRI orientations for an example PLI slide, demonstrating good agreement of the in-plane BAS and microscopy orientations and thus validating our approach.

#### 2.4.2 Hybrid fibre orientation distributions (FODs)

Our method generated a hybrid fibre orientation per microscopy pixel. To summarise the hybrid orientations within a 3D voxel, these orientations were combined over a local 3D neighbourhood (a voxel) into a fibre orientation distribution (FOD) similar to those from diffusion MRI. The spatial resolution of the voxel (a user-defined, largely arbitrary value) decided the size of the local neighbourhood over which orientations were combined (Supplementary Figure 2). As such, the same hybrid orientations could be combined into FODs at multiple spatial resolutions. In this work, FODs were reconstructed at the spatial resolution of 1.0, 0.6, and 0.4 mm isotropic. Voxels of the required spatial resolution were first defined in MRI space. The hybrid orientations within the local neighbourhood of each voxel were then combined into a 3D frequency histogram of orientations for 256 evenly spaced points across a sphere. Spherical harmonics of order 8 were fitted to the histogram. This order was chosen to make our results most comparable with other existing datasets where order 8 is usually the default. However, higher order spherical harmonics could be used. dMRI-derived (CSD) FODs are typically not normalised for the integral to sum to one. To ensure our hybrid FODs had amplitudes similar to those from dMRI (i.e. to make the tractography outputs comparable), the hybrid FODs were scaled on a voxel-wise basis so that the amplitude of the maximum hybrid/CSD FOD peak was equivalent. This scaling method was chosen based on the visual evaluation of comparable outputs from the hybrid and diffusion tractography. Alternative approaches may involve aligning the mean amplitudes of the hybrid and dMRI FODs by equating the first spherical harmonic coefficient, corresponding to the *l* = 0, m = 0 term.

In BigMac, there was a region in the centre of the brain (approx. 1.8–3 mm thick) with missing microscopy because the brain was sectioned in two halves (an anterior and posterior block). In this region, the missing data were filled with the FODs from the dMRI data estimated via CSD (Tournier et al., 2007) (Supplementary Figure 3). To fill the missing data of the hybrid orientation at 0.4 mm and 1 mm, the CSD FODs were up-/down-sampled as appropriate with MRtrix3 (Tournier et al., 2019).

#### 2.4.3 Gelatine artefact

In certain most anterior PLI sections, background birefringence is observed both inside and outside of the tissue that varies slowly across the slide (Supplementary Figure 4). This is due to the anterior PLI slides being coated in a small amount of gelatine which aids the mounting of tissue sections onto the glass slides but which is also birefringent (Tobolsky, 1955). The posterior PLI slides were mounted on plain glass slides without gelatine and so do not suffer from this artefact. In the affected slides, orientations from the grey matter are unreliable, though orientations from the highly myelinated white matter appear reasonable, as the birefringence of myelin appears to dominate (Menzel et al., 2015). When working with the hybrid FODs, it would be possible to mask out the affected regions and replace them with FODs from CSD, or use hybrid orientations from another microscopy (e.g. Gallyas staining that does not suffer from this artefact). We have not implemented that here as our results primarily focus on white matter reconstruction that does not primarily include these grey matter regions.

### 2.5 Experiments

#### 2.5.1 Tractography

Tractography was performed using MRtrix3 and FODs either derived from the hybrid method above (hybrid tractography), or from CSD (Tournier et al., 2007) of the dMRI data (diffusion tractography). Whole-brain tractography was achieved using the iFOD2 algorithm (Tournier et al., 2010) with the following parameters: 30 seeds per voxel in the white matter, step size of 0.2 mm, cut-off value of 0.05, maximal length of 120 mm, and minimal length of 5 mm. Fibre bundle segmentation was performed with inclusion and exclusion masks from XTRACT to extract 42 tracts spanning the whole brain, including association, commissural, limbic and projection fibres (Warrington et al., 2020). The masks were warped from standard (F99) space to the BigMac diffusion space (Van Essen and Dierker, 2007).

**Fig. 3:**
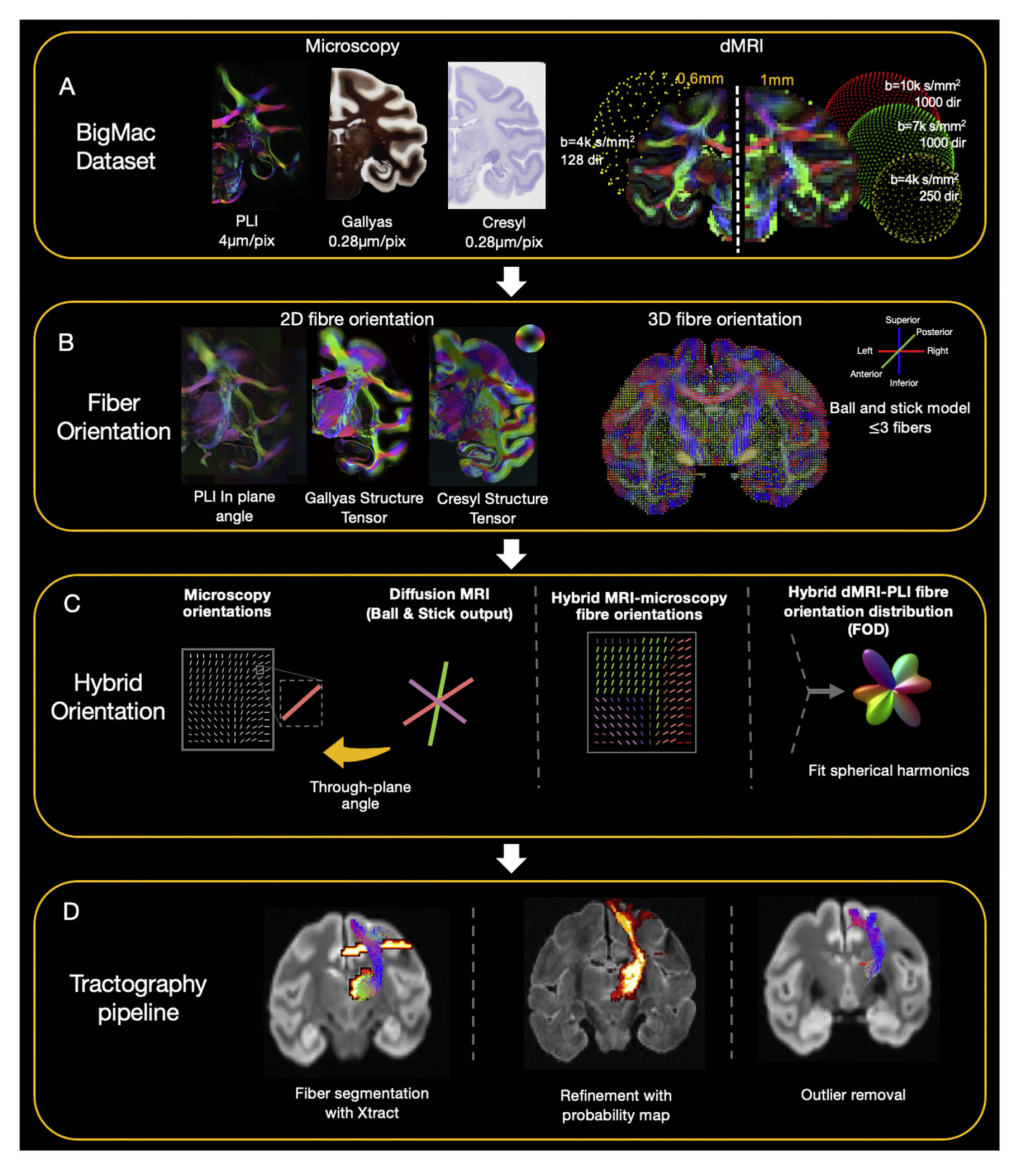
An overview of the analysis pipeline. A) BigMac includes co-registered microscopy (PLI, myelin- and Nissl-staining) and postmortem MRI (dMRI/structural). B) Fibre orientations are extracted from each microscopy contrast (in-plane angle from PLI, structure tensor analysis from histology) and dMRI (Ball and Stick model). C) Hybrid orientations are generated with the in-plane orientation from microscopy and through-plane orientation from dMRI. D) The tractography results are optimised with XTRACT probability maps and outlier removal.

The reconstructed tracts were further refined to remove the spurious streamlines (Fig. 3). First, each streamline within a given tract was compared to the tract probability map from the population-based XTRACT white matter atlas obtained from 6 macaque brains (Warrington et al., 2020). The streamline was assigned a score based on the mean probability value of the voxels the streamline intersected. If the mean probability of a streamline was lower than 0.2, it was discarded (Yeatman et al., 2012). Second, streamlines that were 4 standard deviations longer than the mean fibre length or deviated over 5 standard deviations relative to the core of the fibre were removed. The parameters were chosen based on the literature and were fine tuned for our data (Yeatman et al., 2012). The density map of each tract was calculated on a voxel-wise basis as the number of streamlines per voxel divided by the total number of streamlines of the tract.

#### 2.5.2 Gyral bias

Gyral crowns were defined as regions of high convexity and gyral walls were defined by their low curvature. FODs near the gyri and gyral streamline structure were examined for different spatial resolutions (0.4, 0.6 and 1 mm). Whole-brain tractography was then performed with the parameters mentioned above, but now with 5 seeds per voxel to facilitate easier visualisation of streamlines near the cortex.

#### 2.5.3 Bottleneck regions

We investigated two bottleneck regions within the motor network to determine whether hybrid tractography with different spatial resolutions can retain the topographic organisation of fibre bundles when passing through the bottleneck regions. First, we investigated whether the topographic medial-lateral representation of the trunk, arm and face areas across the motor cortex would be retained for fibres projecting through the internal capsule, as has been previously described (Kretschmann, 1988; Morecraft et al., 2002; Park et al., 2001). Second, we studied fibre bundles projecting from the primary motor and somatosensory motor cortex into the brainstem to determine whether the anterior-posterior distribution was retained (Asan et al., 2022).

The ROIs in the motor/somatosensory cortex were manually drawn on the structural MRI surface and then converted into volume space, warped to the dMRI space and used for tractography seed masks. The internal capsule mask was obtained from the D99 atlas v2.0 (Saleem et al., 2021) and the brainstem mask was obtained from the subcortical atlas of the Rhesus Macaque (SARM) (Hartig et al., 2021). Both were warped to BigMac diffusion space. Seeding from the ROIs in the cortex, tractography was performed following the same parameters as above. Only streamlines going through the internal capsule or brainstem were retained. Tract density maps were generated for each ROI. The masks within the internal capsule and brainstem were colour-coded based on the ROI with the highest number of streamlines. The outputs in the internal capsule and brainstem were compared for both hybrid orientations and dMRI data.

To identify the bottleneck area, fixel-based analysis was performed with MRtrix3 (Tournier et al., 2019). A fixel describes a specific fibre population within a voxel. Tracts generated from each seed ROI were converted to fixel density maps using the tck2fixel command. Here the FOD was first segmented into discrete fixels and each streamline passing through a voxel was assigned to the fixel that exhibited best alignment (Dhollander et al., 2021). The fixel density map was then thresholded at 5% and converted into a fixel mask. The bottleneck area was identified as the region where multiple fibre bundles use the same fixel when fibre tracking through the voxel (“multi-bundle fixels”).

#### 2.5.4 Comparison with anatomic tracers

We compared our hybrid tractography to a weighted connectivity matrix derived from retrograde tracer data, which serves as a ground truth estimate of structural connectivity (Donahue et al., 2016; Markov et al., 2012, 2010). We used a weighted connectivity matrix constructed from tracer injections in 28 monkeys to examine the connectivity profiles of cortical-cortical pathways which is openly available on the Brain Analysis Library of Spatial maps and Atlases (BALSA) database (Van Essen et al., 2017). Tracers were injected into 29 regions of 91 cortical areas from the macaque atlas in the left hemisphere (Markov et al., 2010; Paxinos et al., 2024; Saleem and Logothetis, 2012), and the fraction of labeled neurons (FLNe), defined as the number of labeled neurons in each cortical area relative to the total number of neurons in the injection, was used to quantify connectivity. As we aimed to investigate the improvement of hybrid tractography over dMRI tractography with the tracer connectivity as a reference, we applied a threshold of 0.0001 to exclude very sparse tracer connections that are unlikely to be captured by tractography (Markov et al., 2012). The cortical atlas was co-registered to BigMac space and hybrid/diffusion-only tractography was seeded from the 29 injection sites of the left hemisphere with the tractography parameters mentioned above. The number of streamlines projecting to each of the 91 cortical regions, divided by the total number of streamlines from that injection site, was used to construct the tractography connectivity matrix (29 × 91 areas). The connectivity matrix was generated for hybrid tractography at 0.4 mm, 0.6 mm and 1 mm.

To facilitate quantitative comparison via a ROC curve, the tracer matrix was first binarised using a threshold of 0.0001. The hybrid/diffusion-only connectivity matrices were then also binarised using thresholds ranging from 0 to 0.25 and the number of true positives (TP), false positives (FP), true negatives (TN) and false negatives (FN) was counted for each threshold. The sensitivity was calculated as the true positive rate TPR=TP/(TP+FN) and specificity as the true negative rate TNR=TN/(TN+FP). The ROC curve was then plotted as sensitivity versus 1-specificity. The AUC value was calculated using MATLABs (2021a) perfcuve function. Overlap matrices (hybrid/diffusion tractography versus tracer) were then plotted for different tractography thresholds with the tracer in yellow, tractography in blue, and overlap in green. The correlation between tractography and tracer connectivity weights was evaluated by plotting the data on a logscale to quantitatively assess the predictive power of tractography. Tracer data were thresholded at 1e-6 and tractography data were thresholded at 1e-4. The Pearson correlation coefficient was calculated and reported.

## 3 Results

Note, we use the term MRI-microscopy to refer to the general method, as multiple microscopy contrasts can be used to create hybrid orientations. MRI-PLI is used to denote the hybrid orientations reconstructed using PLI which estimates the in-plane fibre orientation based on tissue birefringence (Axer, 2011; Axer et al., 2001, 2011; Larsen et al., 2007).

### 3.1 Generating hybrid MRI-PLI fibre orientations

**Fig. 4:**
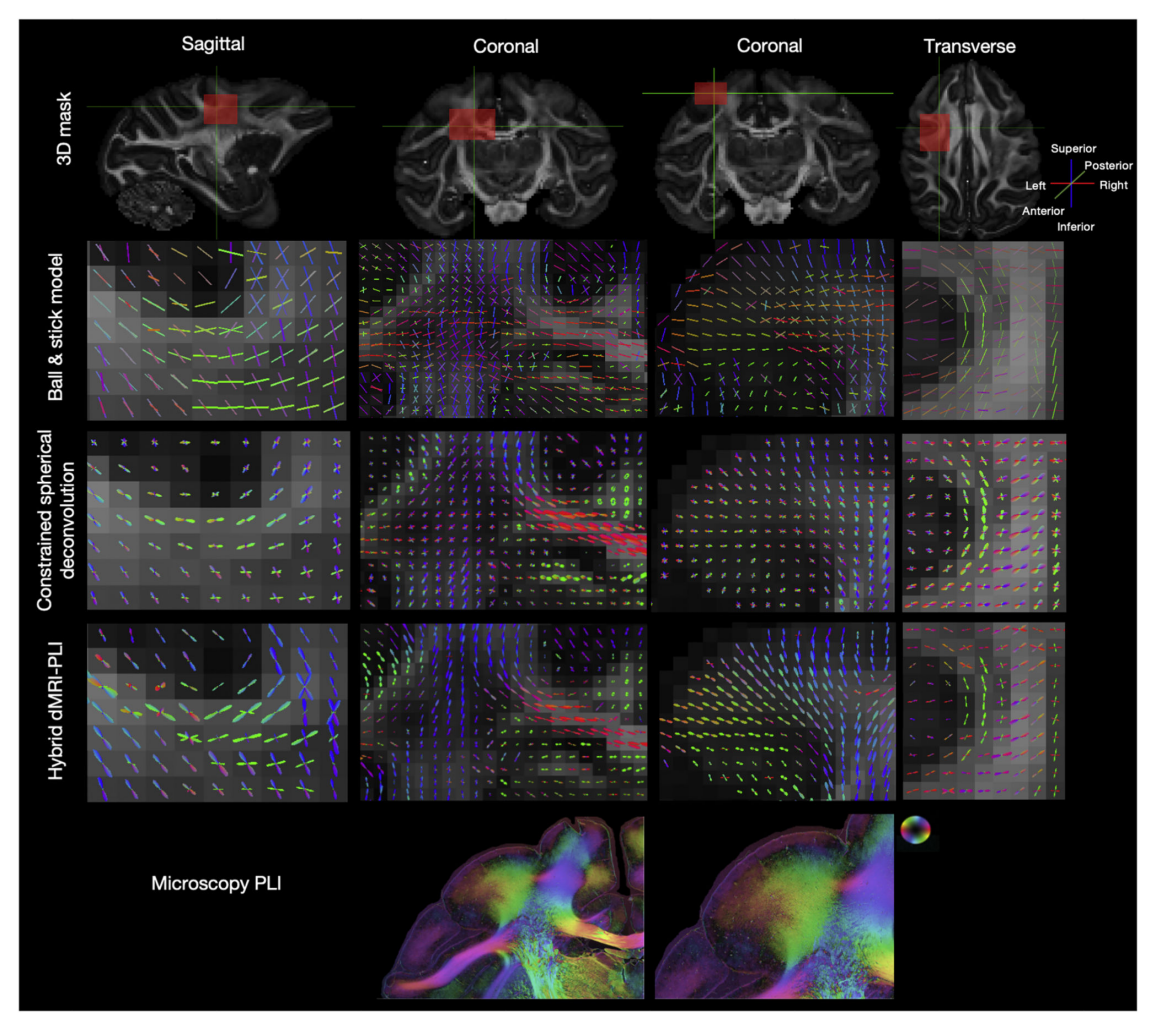
Comparison between dMRI FOD and hybrid MRI-PLI FOD. The FOD generated from the Ball and Stick model (Top), constrained spherical deconvolution (Middle) and hybrid MRI-PLI (Bottom). The orientations in sagittal, coronal and transverse views are presented for U-fibres (left and right), a region covering the corpus callosum and centrum semiovale (middle left) and cortex (middle right). The PLI hue-saturation-value with the colour-coded orientations is provided at the bottom, and the contrast has been edited to highlight the grey matter. Note the colour schemes for the FODs and PLI are not equivalent.

Hybrid MRI-microscopy orientations were estimated for each microscopy pixel and then combined over a local neighbourhood to create fibre orientation distributions (FODs). Fig. 4 compares hybrid FODs at 0.6 mm isotropic to those obtained from dMRI using both the Ball and Stick model and constrained spherical deconvolution (CSD) where the latter also uses spherical harmonics making the FODs easy to compare. For the hybrid FODs, the microscopy mostly informed the orientations within the coronal plane, whilst the dMRI provided through-plane information in the sagittal and transverse views. We observe how the hybrid MRI-PLI FODs agree with the fibre orientations from Ball and Stick and CSD in all three views and faithfully depict U-shaped fibres connecting cortical areas between adjacent gyri. The fibre orientations are generally well aligned and follow expected anatomy, giving confidence in the fidelity of the hybrid outputs. This is true both for fibre orientations that lie primarily within the microscopy plane, such as the corpus callosum, and those that are primarily through the microscopy plane, such as the cingulum. The hybrid FODs are mostly narrower than CSD, indicating lower fibre dispersion (Howard et al., 2019). Further, though crossing fibre populations are depicted in the hybrid FODs, they are less frequent, and the hybrid method estimates fewer, more dominant fibre populations compared to CSD. This is particularly evident at the white matter-grey matter boundary where the hybrid MRI-PLI FODs show a more anatomically accurate pattern of fibres fanning into the cortex as frequently described in myelin-stained histology. We also observe fewer crossing fibres within some, but not all, white matter regions, including in parts of the centrum semiovale.

One important feature of our hybrid method is that the hybrid FODs can be constructed at different resolutions from the same underlying data by changing the size of the combined neighbourhood region (Supplementary Figure 5). This allows us to investigate how spatial resolution affects tractography without confounding factors such as signal-to-noise ratio changes in MRI acquired at different resolutions.

Supplementary Figure 5 demonstrates how using 0.6 mm resolution postmortem dMRI and PLI microscopy at 4 μm/pixel we created hybrid FODs at multiple resolutions: 1 mm isotropic, 0.6 mm isotropic, 0.4 mm isotropic, and 0.1 × 0.4 × 0.1 mm. In the latter, the through-plane resolution (0.4 mm) was determined by the distance between consecutive PLI slices (0.35 mm).

### 3.2 Whole-brain hybrid MRI-PLI tractography

**Fig. 5:**
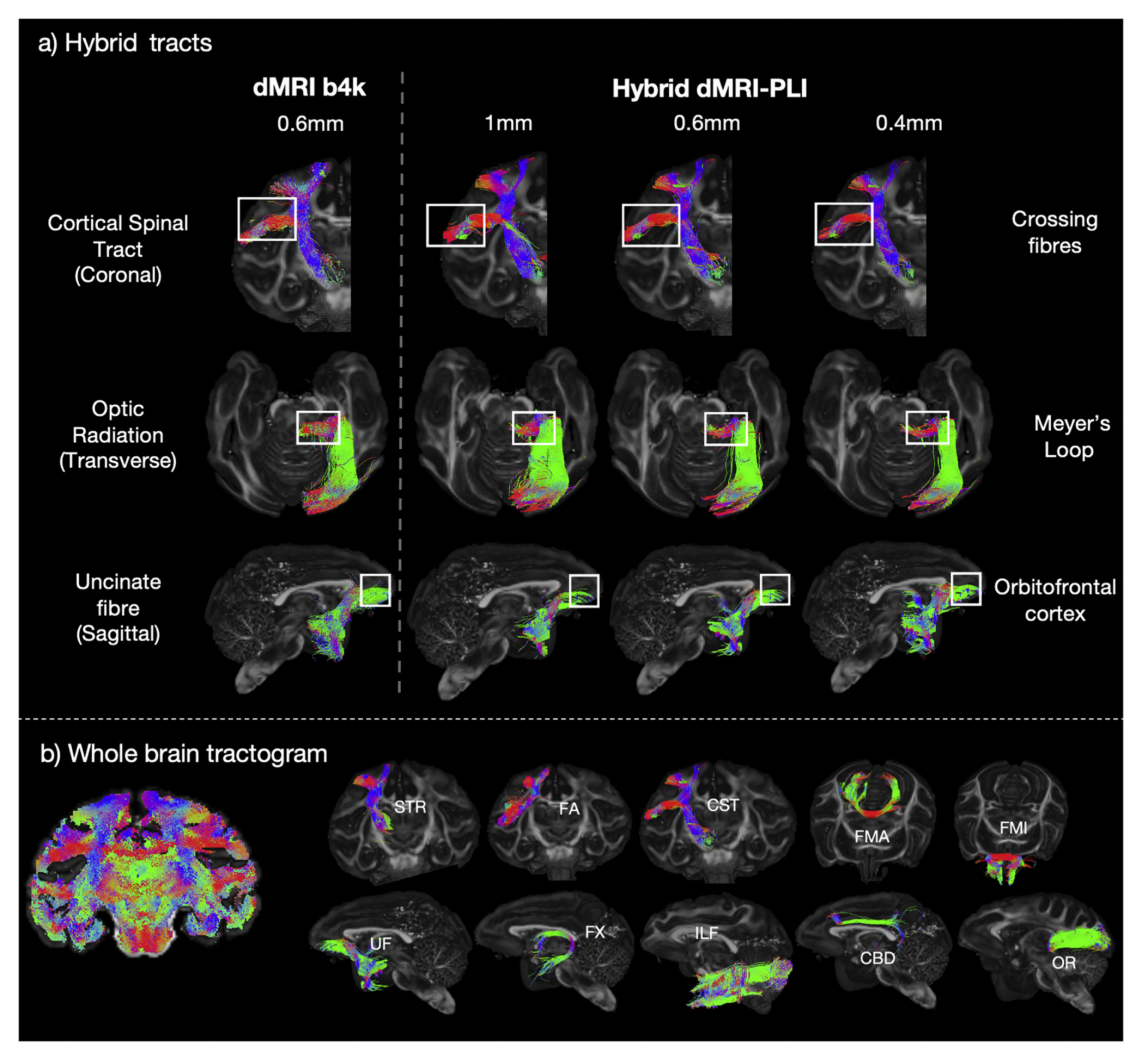
Tracts generated using the hybrid tractography. a) The hybrid method can successfully reconstruct tracts throughout the brain including the corticospinal tract, an example tract within the coronal plane where microscopy is the most informative (Top), the optic radiation, a tract extending primarily along the anterior-posterior axis where dMRI provides most information (Middle), and the uncinate fibre (Bottom), a tract connecting the anterior temporal lobe and the orbitofrontal cortex. The anatomical features of interest are labelled with white boxes. The background anatomical image is the fractional anisotropy behind the tracts for visualisation. b) A whole-brain tractogram is shown. Ten example tracts generated from the hybrid tractography at 0.6 mm isotropic are illustrated including the superior thalamic radiation (STR), frontal aslant (FA), corticospinal tract (CST) in the coronal view, forceps major (FMA), forceps minor (FMI) in the axial view, uncinate fasciculus (UF), fornix (FX), inferior longitudinal fasciculus (ILF), cingulum subsection: dorsal (CBD), optic radiation (OR) in the sagittal view.

Using fibre orientation estimates from the hybrid MRI-PLI, fibre bundles were reconstructed with a standard tractography pipeline (Fig. 3). Fig. 5 displays the reconstruction of three example tracts at the hybrid spatial resolutions of 0.4 mm, 0.6 mm, and 1 mm isotropic, alongside tractography using conventional dMRI-only analysis (CSD) at 0.6 mm. We specifically chose tracts that represent two extremes with respect to how the hybrid orientations relate to the underlying data: (i) the cortico-spinal tract predominantly runs in parallel to the microscopy plane such that microscopy is most informative; (ii) the optic radiation extends through the microscopy plane for which dMRI is most informative. Hybrid and dMRI tractography produce similar reconstructions of both tracts. Both tracts are characterised by intricate anatomical features: the cortico-spinal tract tracks through multiple crossing fibre regions, with branches extending to both superior and lateral regions of the motor cortex; the optic radiation contains a backward curving structure known as Meyer’s Loop in the anterior portion (Chamberland et al., 2018). The hybrid method successfully reconstructs both complex structures. The uncinate fibre tract represents a case where the hybrid tractography does not perform so well. Most of the uncinate fibre can be robustly reconstructed with a clearly observed U shape structure extending from the anterior temporal lobe to the orbitofrontal cortex.

However, the hybrid method fails to capture the most anterior part of the uncinate tracking through the orbitofrontal cortex, which is well reconstructed using dMRI-tractography. The limited anterior extent may be caused by registration inaccuracies due to the limited amount of brain tissue and less distinct anatomical features to drive the registration, or affected by the background gelatine in the most anterior part of the brain (c.f. 2.4.3 Gelatine artefact). The latter can likely explain differences in the tracts cortical terminations, rather than the tract core, as background gelatine was shown to predominantly affect PLI in the grey matter.

Having established that our method can provide reliable tract reconstructions, we then proceeded to reconstruct fibre bundles spanning the whole brain (Fig. 5b & Supplementary Figure 6). The XTRACT toolbox was employed to define seed and target regions of interest (ROIs) for a total of 42 tracts, comprising association, commissural, and projection fibres (Warrington et al., 2020).

The BigMac macaque brain was found to have a cerebral bleed in the right hemisphere, as observed in the MRI (loss of signal) and microscopy (missing tissue). This bleeding site primarily affected the grey matter but had some overlap with tracts including SLF2. Consequently, we observed asymmetry in the reconstruction of SLF2 between the left and right hemispheres (Supplementary Figure 7), where streamlines failed to track through the bleeding site on the right. Despite this limitation, other tracts were successfully reconstructed using the hybrid orientations, yielding anatomically expected structures (Fig. 5b). The results further demonstrate that our method is applicable to a wide range of major white matter tracts and can reconstruct tracts spanning the whole macaque brain.

### 3.3 Hybrid tractography resolves the gyral bias at high resolution

**Fig. 6:**
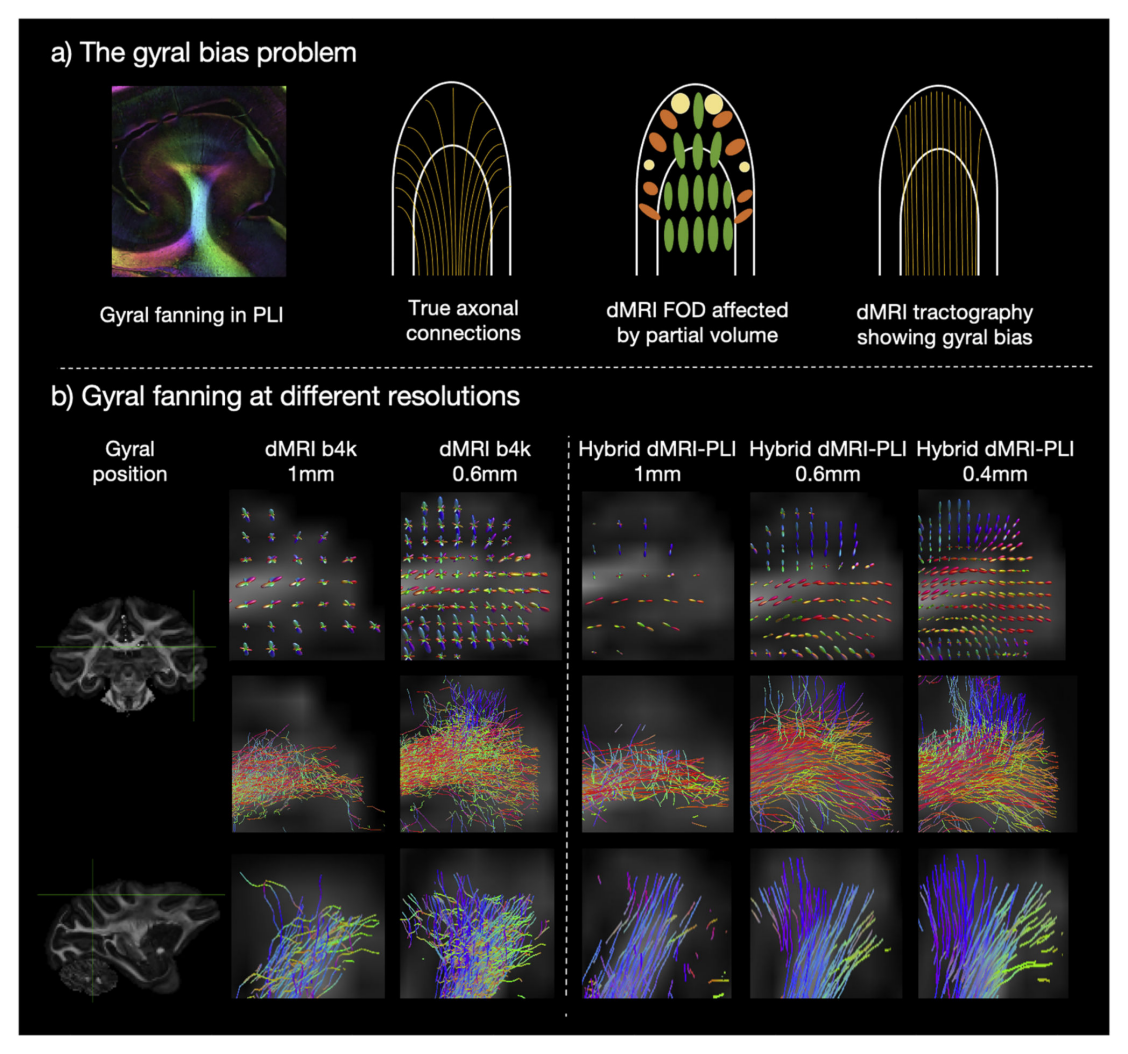
High resolution hybrid tractography eliminates the gyral bias. a) Gyral bias. Diffusion tractography trajectories (streamlines) predominantly terminate at the gyral crowns but fail to turn into the gyral wall. The ground truth gyral fanning is demonstrated using PLI and a schematic representation of fibre connections. b) Gyral fanning at different resolutions. FODs (Top) and tractography streamlines of two gyri which primarily lie within (Middle) and through (Bottom) the microscopy plane. Outputs are shown for dMRI and hybrid MRI-PLI reconstructed at different isotropic resolutions. The gyral bias problem is present in the 1 mm, whilst the high spatial resolution of 0.4 mm successfully delineates the expected fibre fanning.

At the spatial resolution available to dMRI scans, the signal in voxels near the cortex is dominated by fibres projecting towards the gyral crowns. This causes streamlines to terminate at gyral crowns rather than accurately capturing sharp turns into gyral walls, leading to biases in connectivity mapping (Fig. 6). This so-called gyral bias is in part due to the spatial resolution of dMRI (Schilling et al., 2017; Tendler et al., 2022). Our hybrid method provides microscopy-informed fibre orientations that can be reconstructed at multiple spatial resolutions down to the near-native microscopy resolution. We investigated whether this approach can reduce or eliminate the gyral bias problem. In Fig. 6, the gyral bias is evident in the 1 mm hybrid data, with fibres preferentially terminating at the gyral crown. However, with increased spatial resolution we observe an increasing number of streamlines extending towards the gyral walls, aligned with previous observations from microscopy (Tendler et al., 2022). The dMRI-only data shows similar trends of reduced gyral bias with increased spatial resolution, though with a less clean fanning pattern than that observed in the hybrid results. These results suggest that increasing the spatial resolution in both diffusion and hybrid tractography can overcome the gyral bias problem which is consistent with previous findings (Schilling et al., 2017; Tendler et al., 2022).

### 3.4 Hybrid tractography preserve topography in bottleneck areas

**Fig. 7:**
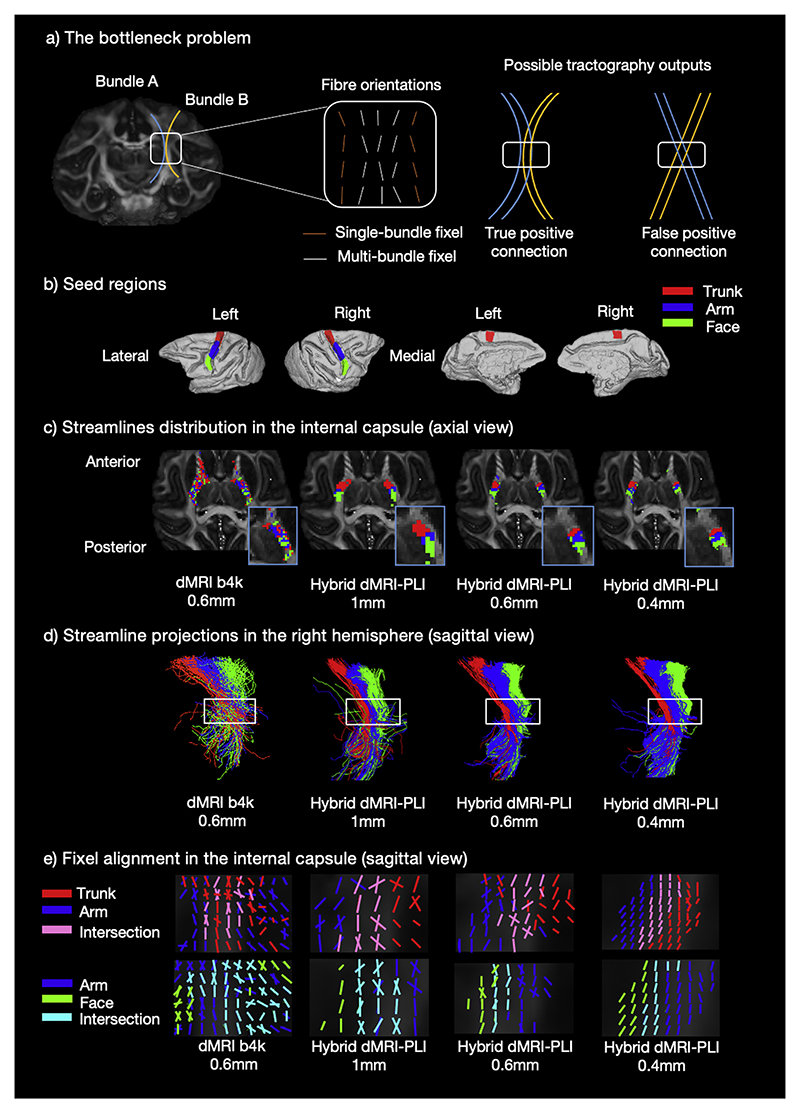
Hybrid tractography preserves topography in the internal capsule. a) Bottleneck problem. An example in the internal capsule (IC) is shown (Adapted from Schilling et al., 2022). Two fibre bundles, originating and terminating at different locations converge with similar orientations within the bottleneck region (truth axonal connection). Fixels (fibre orientations per voxel) are classified based on the number of fibre bundles passing through each fixel: multi-bundle fixels (white) have multiple associated fibre bundles and single-bundle fixels have a single associated fibre bundle (orange). The bottleneck region, identified as the multi-bundle fixel where streamlines become indistinguishably mixed, generates false positive connection. As a result, a single FOD pattern can produce two probabilistic tractography outputs. b) ROIs relate to the functional representation of the trunk, arm and face regions shown for both medial and lateral parts of the two hemispheres. Tractography was seeded from the ROIs to reconstruct streamlines passing through the IC. c) The density map and (d) streamline projections are shown. The blue box indicates a zoomed region in (c) and the white box in (d) is the bottleneck region of interest. The bottleneck problem is observed in the dMRI as streamlines from the ROIs are mixed. With the hybrid method, the streamlines from each ROI demonstrate a clear anterior-posterior distribution in the bottleneck region. e) Fixel-based analysis was performed to generate a fixel density map for each ROI in the internal capsule. The red, blue and green colours represent fixels associated with the trunk, arm and face regions respectively. Pink shows fixels associated with both the trunk (red) and arm (blue) ROIs. Cyan shows the intersection of fixels from both the arm (blue) and face (green).

Another limitation of dMRI tractography is the bottleneck problem. In bottleneck regions, multiple fibre bundles merge causing streamlines to become indistinguishably mixed at the spatial resolution of dMRI (Maier-Hein et al., 2017; Schilling et al., 2022). Without additional anatomical knowledge, it is difficult to retain information about the origin and termination of individual tracts passing through a bottleneck (Fig. 7a). We further investigated the use of hybrid method in tackling bottleneck regions to ask whether spatial resolution and/or the microscopy-informed fibre orientations can provide superior tract separation than typical dMRI analysis. Our first bottleneck of interest was the internal capsule (IC). This was motivated by the known topographic arrangement of fibres within this tract, mirroring the topography of the motor cortex. The medial-to-lateral functional organisation of the motor cortex has a topographic organisation, which is maintained along the anterior-to-posterior axis of the IC, with fibres originating from medial “trunk (and legs)” regions located anteriorly within the IC compared to those from lateral “face” regions (Kretschmann, 1988; Morecraft et al., 2002; Park et al., 2001). Streamlines were seeded from three ROIs defined on the precentral gyrus representing the trunk (and legs), arm and face regions with a medial-to-lateral distribution (Fig. 7b). Fig. 7c shows voxels in the IC colour-coded by the ROI with the highest streamline density. Fig. 7d visualises the streamlines from each ROI projecting through the IC. Whilst dMRI mixes streamlines from distinct ROIs as they pass through the IC, the hybrid method consistently retains the strong topographic organisation of streamlines through the bottle-neck region, where the order of trunk-arm-face regions along the anterior-posterior axis in the IC follows neuroanatomical expectations (Kretschmann, 1988; Morecraft et al., 2002; Park et al., 2001).

Fixel-based analysis was then used to visualise the fibre populations in each voxel associated with each tract (Dhollander et al., 2021), quantifying tract orientation overlap within the bottleneck (Supplementary Figure 8). In each voxel, the FOD was divided into discrete fibre populations, or “fixels”. During tractography, each streamline passed through a voxel by following a specific fixel. Consequently, we can describe each streamline as a series of fixels projecting from our seed ROI. Combining across all streamlines in a given tract produced a fixel density map. This is analogous to a streamline density map, but now on a fixel rather than voxel basis. The density map was then converted into a tract-specific fixel mask using a 5 % threshold.

Regions where a single fixel is transversed by multiple fibre bundles (referred to as a “multi-bundle fixel”) are defined as bottleneck regions. Conversely, fixel associated with a single bundle is termed “single-bundle fixel” (Schilling et al., 2022). In Fig. 7e, single-bundle fixels are colour coded using red, blue and green according to the ROI from which they are seeded (red=trunk, blue=arm, green=face). Multi-bundle fixels exhibit overlapping colours such as pink resulting from the overlap between the red (trunk) and blue (arm) fixels, or cyan resulting from the overlap between blue (arm) and green (face) fixels. In the dMRI analysis, we observe a large number of multi-bundle fixels (i.e. a large bottleneck region). This suggests that streamlines originating from different motor cortex ROIs tend to jump from one tract to the other. In the hybrid results, there is a clear separation of the ROIs on either side, with an intersecting bottleneck region in the middle where the two pathways run parallel to each other with shared orientations. This pattern is most evident in the 0.4 mm, as with increased spatial resolution, the bottle-neck region decreases in size and the separation becomes clearer.

Together, the results demonstrate how, irrespective of the spatial resolution, microscopy-informed tractography mitigates the bottleneck problem in the IC. Interestingly, this holds even for the 1 mm hybrid reconstruction, a coarser resolution than that of the dMRI data used in this comparison (0.6 mm).

**Fig. 8:**
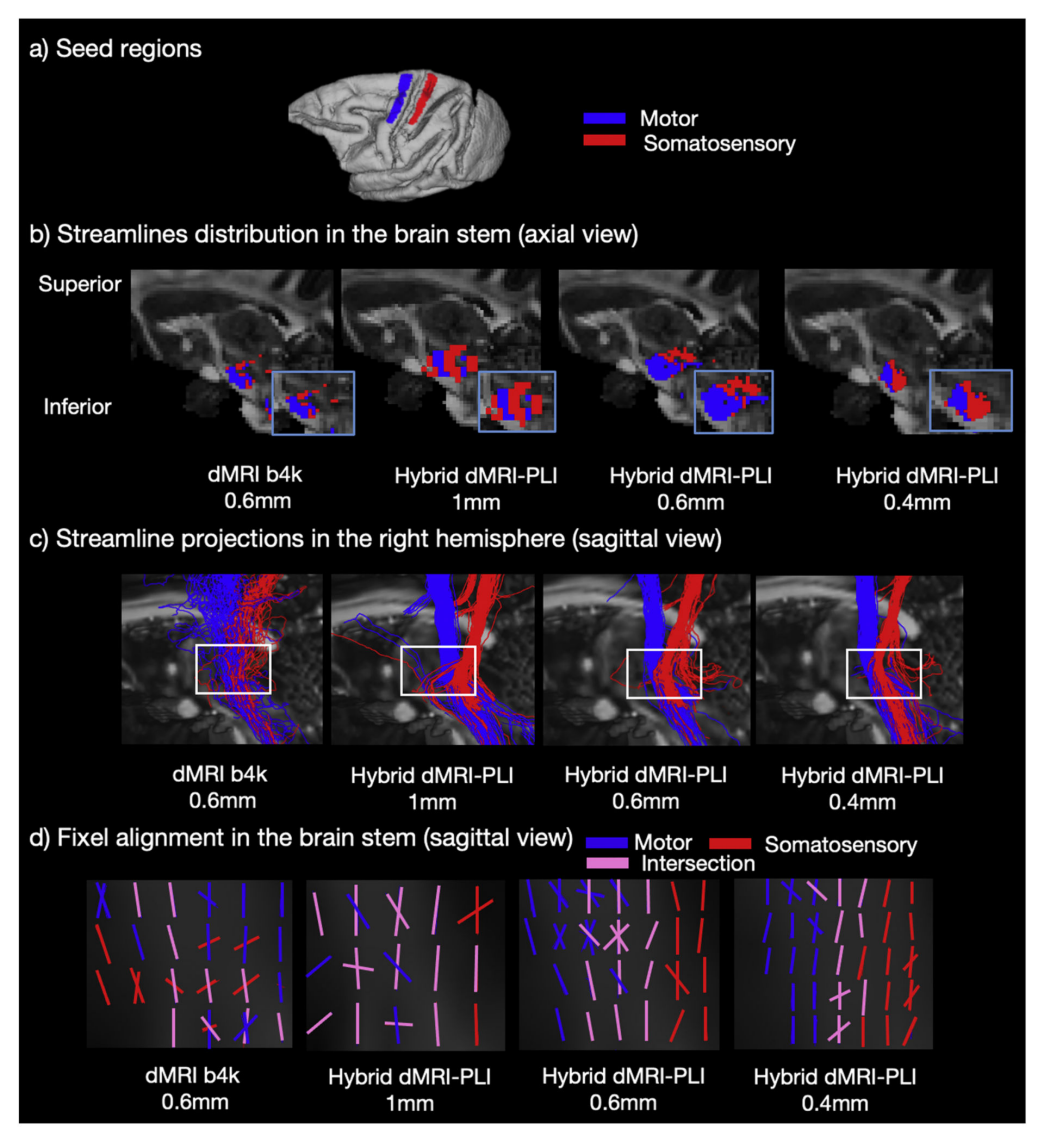
Hybrid tractography preserves topography in the brainstem. a) ROIs in the primary motor (blue) and somatosensory (red) cortex. Tractography was seeded from the ROIs to reconstruct streamlines passing through the brainstem. b) The density map in sagittal view and (c) the streamline projections are shown. The blue box indicates a zoomed region in (b) and the white box in (c) is the bottleneck region of interest. The bottleneck problem is observed in the 0.6 mm dMRI and 1 mm hybrid method as streamlines from the two ROIs are mixed. In the hybrid method at higher resolution (0.6 and 0.4 mm), the streamlines from each ROI demonstrate a separable distribution in the brainstem. d) Using fixel-based analysis, fixels from primary motor cortex (blue), somatosensory cortex (red), and overlapping fixels (pink) are shown.

A second bottleneck region in the brainstem was studied using tractography seeded from ROIs in the primary motor and somatosensory cortex (Fig. 8) (Asan et al., 2022). As above, density maps and tractography results are presented for dMRI and hybrid orientation at different spatial resolutions. The bottleneck problem is observed in both the dMRI and the hybrid method at 1 mm as streamlines from the two ROIs are mixed. In the hybrid high resolution results (0.4 mm and 0.6 mm), the streamlines from each ROI demonstrate a separable distribution. Streamlines from the primary motor ROI are observed to be more anterior to those from the somatosensory ROI, mirroring the anterior-posterior topographic organisation of the cortex. As in the IC, the fixel-based analysis indicates how at higher spatial resolution there are fewer fixels associated with the bottleneck (pink), facilitating better tract separation.

### 3.5 Comparison between hybrid tractography and tracer data

**Fig. 9:**
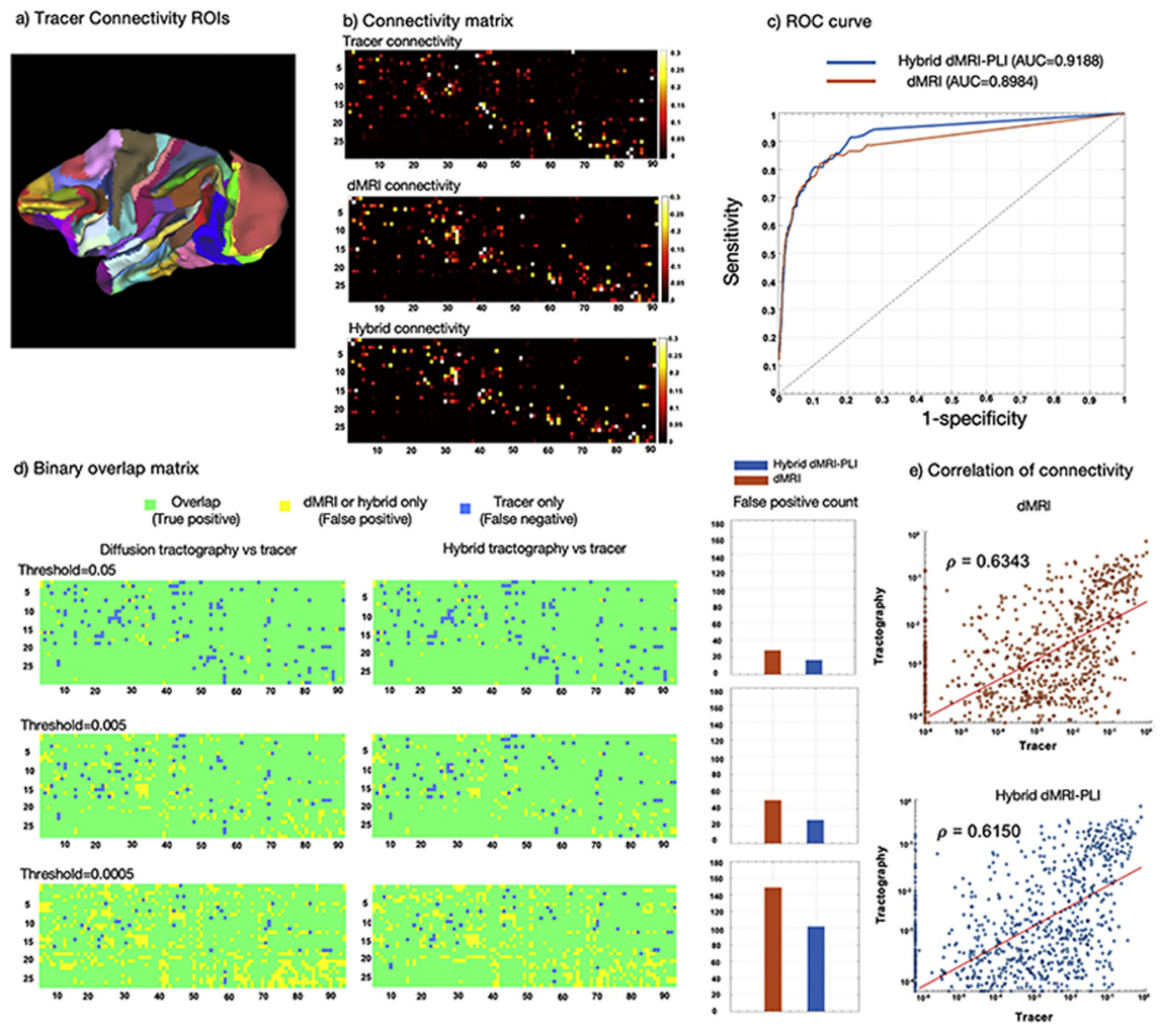
Comparisons of 0.6 mm hybrid and dMRI tractography to tracer data. a) The 91 cortical areas of M132 surface parcellation are shown for the left hemisphere. b) Weighted matrices of size 29 × 91 are shown for the tracer, dMRI tractography, and hybrid tractography. c) An ROC curve illustrating the specificity and sensitivity of hybrid/diffusion-only tractography relative to the tracer connectivity. Different thresholds were applied to the tractography ranging from 0 to 0.25 and the true positive and false positive rates were calculated. d) Binary overlap matrices for different tractography thresholds where tracer only connections are represented in blue (false negative), tractography only in yellow (false positive), and overlap in green (true positive and negative). False positive connectivity was counted for dMRI and hybrid tractography. e) Scatter plot comparing tracer data with tractography (both dMRI and hybrid dMRI-PLI). Tractography data were thresholded at 1e-4 and tracer data were thresholded at 1e-6. Correlations were investigated for several different threshold for tractography (0.0005, 0.005, 0.05), producing similar results (data not shown). The red line denotes the least absolute residual fit, and *ρ* is the Pearson correlation coefficient. Data points on the x- and y- axis (*x* = 0 or *y* = 0) were excluded from the correlation analysis.

To investigate the neuroanatomical accuracy of hybrid MRI-PLI tractography, Fig. 9 uses a tracer-based connectivity matrix as an estimation of ground truth structural connectivity (Donahue et al., 2016; Markov et al., 2012, 2010). Fig. 9b visually compares the normalised connectivity matrix from the tracer to those generated from dMRI and hybrid MRI-PLI tractography at 0.6 mm. As visual comparisons are difficult, we then binarised the tracer matrix using a threshold of 0.0001 and generated a receiver operating characteristics (ROC) curve to quantitatively evaluate the sensitivity and specificity of tractography in detecting brain connectivity relative to the tracer (Fig. 9c). The hybrid method shows larger AUC (area under the ROC curve) with superior sensitivity compared to dMRI for the same level of specificity, and superior specificity for the same level of sensitivity, except for when specificity is very high and sensitivity is low where the methods become similar. Fig. 9d shows overlap matrices between diffusion/hybrid tractography and tracer for different tractography thresholds. Tracer, tractography, and overlap are visually represented using distinct colours, allowing visualisation of false positive, false negative, and true positive connections. At lower thresholds, the hybrid method demonstrates fewer false positive connections compared to dMRI tractography. Fig. 9e correlates the tracer and tractography connection strengths, with similar correlation coefficients for the hybrid method (ρ = 0.6150) compared to dMRI (ρ =0.6343) both of which are comparable to the correlation coefficient reported in previous studies (Donahue et al., 2016). Similar results are found for hybrid MRI-PLI tractography at 1 mm and 0.4 mm (Supplementary Figure 9). These results suggest the hybrid method demonstrates neuroanatomical accuracy in estimating brain connectivity above that of typical diffusion tractography.

### 3.6 Hybrid MRI-microscopy can be performed with various microscopy contrasts

**Fig. 10:**
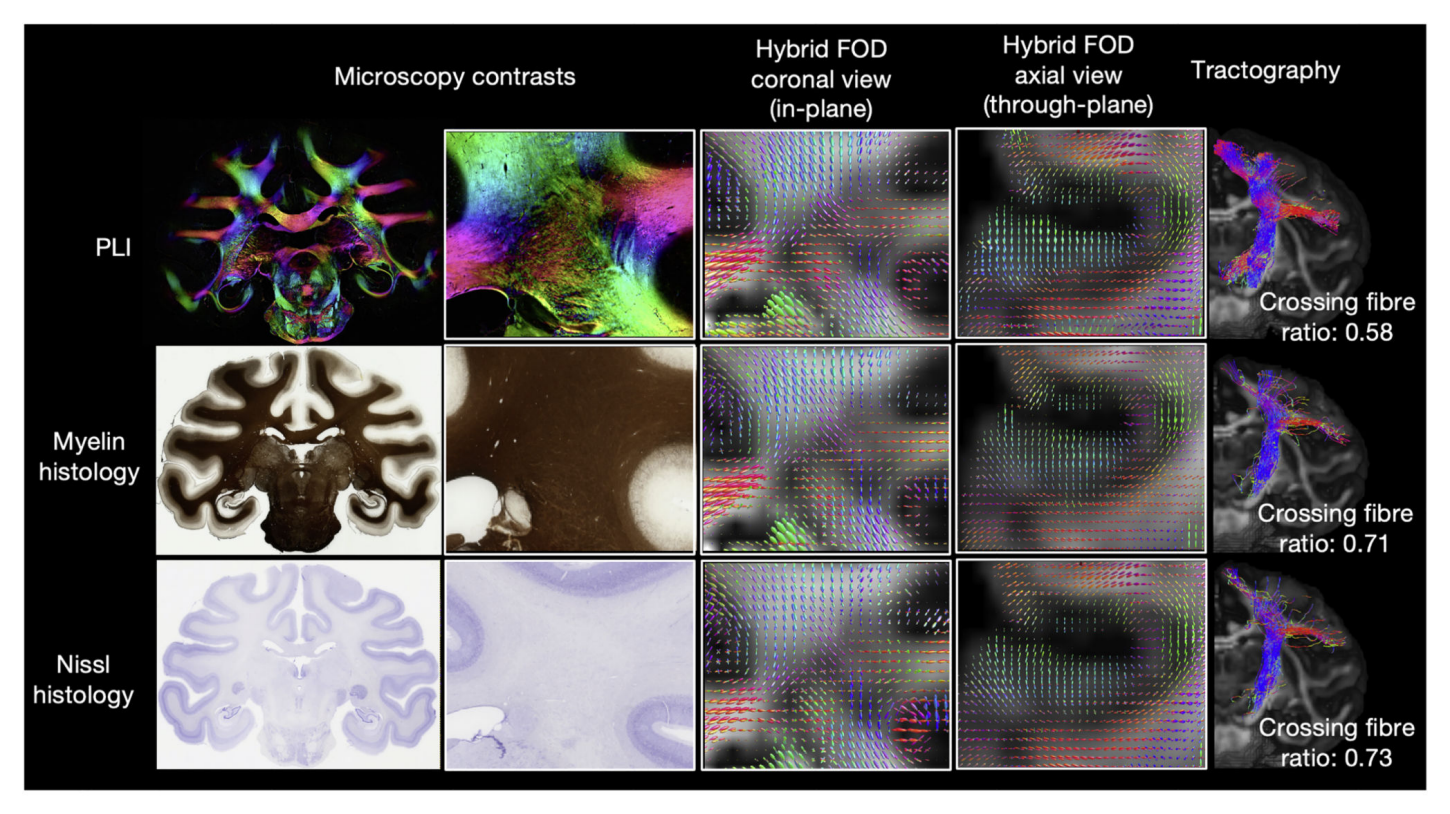
Multiple microscopy contrasts can inform hybrid tractography. Hybrid fibre orientation distributions reconstructed at 0.6 mm resolution from different microscopy contrasts: PLI, myelin- and Nissl-stained histology (Gallyas silver/Cresyl violet-stained). All three contrasts show fibre orientations aligned with neuroanatomical expectations. Noticeably, the histology FODs depict more multi-fibre voxels (crossing fibre ratio = *N*_*wm*,*multi−fibre*_/*N*_*wm*,_ _*voxels* *with* *fibre*_). These FODs can be fed into tractography to reconstruct white matter tracts (example corticospinal tract shown).

The hybrid orientations can also be reconstructed from other microscopy contrasts that provide 2D fibre orientation information. Here we demonstrate this using structure tensor outputs from myelin- and Nissl-stained histology also included in the BigMac dataset (Gallyas silver and Cresyl violet stains respectively) (Bigun et al., 2004; Budde and Annese, 2013; Budde and Frank, 2012; Seehaus et al., 2015). Fig. 10 shows how the resulting hybrid FODs at the 0.6 mm isotropic resolution are consistent among microscopy contrasts and follow our neuroanatomical expectations. We observe U-shaped fibres, also known as short arcuate fibres, connecting different gyri for three different microscopy contrasts with a consistent curving structure even in the through-plane orientation, the most challenging orientation for the hybrid method. Interestingly, we also observe notable differences in the hybrid outputs from the different microscopy contrasts, where both myelin- and Nissl-stains estimate considerably more crossing-fibre voxels than PLI, as quantified by the crossing fibre ratio calculated across the whole brain. Nonetheless, all three microscopy contrasts facilitate tractography reconstruction of the corticospinal tract, providing preliminary results on, and giving confidence in, the generalisability of the method. The application of the method to different microscopy contrasts provides opportunities for other multi-model datasets to reconstruct 3D fibre tractography.

## 4 Discussion

We develop a method that facilitates microscopy-informed tract reconstruction across the whole brain without relying on 3D microscopy or invasive tracers. Our method requires data (diffusion MRI and light microscopy) that is readily attainable, meaning our method is translatable across datasets and species, including into humans. Utilising whole-brain, densely-sampled MRI and microscopy data in the macaque, we construct hybrid MRI-microscopy fibre orientations that are both informed by high resolution microscopy and provide 3D information at the whole-brain level. By combining both modalities in our hybrid reconstruction we assigned the through-plane orientation of the BAS samples to their precise sub-voxel localisation and informed the 3D configuration of fibre orientations from the microscopy. By utilising a dMRI fibre reconstruction method that produces multiple fibre estimates per voxel, our hybrid method could “super-resolve” 3D fibre orientations at a resolution higher than the native MRI. We demonstrate how the hybrid orientations can be used by tractography algorithms for neuro-anatomical investigation. Further, we show how the hybrid outputs (FODs and tractography) based on high fidelity microscopy can be directly compared to dMRI equivalents within the same subject, making the hybrid method a valuable resource for the dMRI modelling community. Notably, the hybrid orientations can be reconstructed at different spatial resolutions using the same underlying data, facilitating meaningful tractography comparisons across different resolutions without signal-to-noise confounds. This allows for novel insights into tractography performance that can potentially inspire future algorithms or study design. In addition, the high-resolution and myelin-specific tractography achievable via the hybrid method can provide neuroanatomical insight, such as interesting patterns of axonal organisation within larger white matter tracts, or reconstruction of the myelinated connectome.

Our data fusion method uses microscopy information to drive tractography-based tract reconstruction with minimum reliance on the dMRI. This is advantageous as it enforces the outputs to be maximally informed by and at the high resolution of the detailed microscopy. This mindset is considerably different from previous approaches that aim to determine optimal tractography protocols by first performing tractography, comparing these outputs to microscopy/tracing, and using this comparison for parameter up-date or to study how various parameter combinations or methods influence the tractography sensitivity and accuracy (Yendiki et al., 2022). Microscopy-informed connectome reconstruction of smaller brains can alternatively be achieved from 3D microscopy data alone. However, to date this has not been achieved at the whole-brain level in larger primate or human brains, as 3D microscopy methods are typically restricted to imaging millimetre scale samples (Georgiadis et al., 2020; Masís et al., 2018; Reckfort et al., 2015), and tracers are typically limited to one or two injection sites per animal. This work provides whole-brain, microscopy-informed connectomics in the macaque, a primate model with complex white matter similar to humans. This white matter complexity is important as it means that models trained on this bespoke dataset may benefit in vivo tractography in human brains where microscopy is not available. In comparison, rodent brains have far fewer crossing-fibre voxels, meaning that models trained on rodents may be less suitable for application to humans.

We investigated whether the hybrid method could address two known challenges in dMRI: the gyral bias and bottleneck problem. Existing dMRI model-based solutions to the gyral bias problem often rely on assumed knowledge of fibre architecture over a canonical gyrus which are often obtained from invasive imaging methods such as microscopy, involving separate brains or even species (Aydogan and Shi, 2018; Wu et al., 2020). For example, St-Onge et al. (2018) incorporated cortical surfaces derived from anatomical images to model the sharp turn of fibres, as fibres were constrained to be radial to the cortical surface. Alternatively, Cottaar et al. (2021) modelled fibre orientation using a vector field, constraining the orientations with the anatomical geometry. In comparison, the hybrid method directly uses high spatial resolution microscopy data in the same brain. The results (Fig. 6) suggest the gyral bias can be reduced and the expected pattern of fibre fanning can be realised with improved spatial resolution (Schilling et al., 2022). We observe similar trends in both hybrid and diffusion tractography, though the results are more striking in the hybrid method. This may result from the hybrid fibre orientations being more directly related to myelinated axons which in microscopy follow clear fanning patterns across the gyrus ex vivo. In comparison, dendrites complicate dMRI modelling for fibres projecting into the cortex. Considering the global volume difference between humans and macaques (macaque brain volume is ∼1/12 of the human brain volume), the spatial resolutions we used (1 mm, 0.6 mm and 0.4 mm) are approximately equivalent to the 2.5, 1.5, and 1 mm in humans, roughly corresponding to resolutions achievable in the clinic, in typical research data and among the highest achievable resolution so far. Consequently, the resolution examined here in the macaque may provide guidance on solving some tractography challenges in the human brain.

Our hybrid method showed particularly promising results when applied to bottleneck regions, where the hybrid tractography resolved patterns of white matter organisation that mirrored the topographic organisation of the cortex (Fig. 7). Bottleneck regions – where multiple fibre bundles converge and share the same orientations – are prevalent in the brain and have been shown to affect a large proportion of white matter tracts leading to false positive streamlines (Maier-Hein et al., 2017; Schilling et al., 2022). Existing solutions relying on anatomical priors or microstructural characteristics demonstrate the potential to identify bundle-specific fibre orientations, which have been shown to effectively reduce false-positive streamlines (Schiavi et al., 2020; Wasserthal et al., 2018). For example, Girard et al. (2017) addressed ambiguities in fibre configurations through microstructure modelling, and Rheault et al. (2019) enhanced tracking specificity by integrating both anatomical and orientational priors. However, these approaches remain susceptible to errors in estimating fibre orientations from dMRI data. In contrast, our method directly utilises microscopy data, thereby bypassing microstructural assumptions and model-based limitations to address the bottleneck problem more accurately. First, we investigated the bottleneck problem in the internal capsule, a relatively thin white matter structure where multiple tracts intermingle to pack within a small volume. Our ability to conserve the order of the motor cortex projections along the anterior-posterior axis in the internal capsule was evaluated. The topographic organisation was conserved in the hybrid tractography at all resolutions including 1 mm but lost in the diffusion tractography with 0.6 mm. This implies that our ability to resolve the anterior-posterior pattern of white matter organisation in the internal capsule is related to some specific feature of the hybrid FODs, rather than simple improvements in spatial resolution. For example, the hybrid FODs often have lower dispersion or fewer crossing fibres than the dMRI (likely due to their high specificity to myelin) which may result in streamlines leaving the cortical seed region and following a smooth and continuous fibre bundle instead of jumping between different tracts. Second, we studied the bottleneck region at the brainstem where PLI has been shown to be superior to dMRI when identifying smaller fibre bundles such as pyramidal tracts (Axer, 2011). Our ability to retain the order of primary motor and somatosensory cortex along the anterior-posterior axis of the brainstem was demonstrated with the hybrid method at higher resolutions (0.6 or 0.4 mm), but not in the 1 mm hybrid outputs or diffusion tractography at 0.6 mm. These results suggest that improved spatial resolution combined with our hybrid specificity can again help to resolve the bottleneck problem. Together, hybrid results present a novel way of investigating the bottleneck problem. Our method has the potential to resolve topographic patterns of white matter organisation within major fibre bundles that are currently considered relatively homogeneous or where long-standing hypotheses regarding white-matter topography are currently still lacking empirical evidence (Belyk et al., 2021).

The hybrid MRI-PLI tractography is generally well aligned with that of dMRI, though there are also some noticeable differences. To better understand whether the hybrid outputs better reflect ground truth anatomy, we compare the connectivity of dMRI and hybrid tractography to tracer connectivity data from other macaque brains (Markov et al., 2012). Our analysis reveals that the hybrid method outperformed dMRI tractography in terms of simultaneously achieving sensitivity and specificity, as indicated by the ROC curve (Fig. 9). We hypothesise this may be because the hybrid FOD is based on myelin-sensitive microscopy and so is fairly myelin-specific, whilst the dMRI FOD is influenced by multiple tissue features (e.g. dendrites, glia, and extra-cellular space) (Alexander et al., 2019; Jespersen et al., 2007; Stanisz et al., 1997). Consequently, the hybrid FODs may allow for superior tracking, particularly in regions with considerable partial voluming such as in the cortical ribbon or in and around subcortical structures.

We demonstrate how our method can be applied to different microscopy contrasts, each of which may provide subtly different orientational information (Fig. 10). Here, we observe a lower crossing fibre ratio in the hybrid MRI-PLI FODs, when compared to hybrid FODs reconstructed from either myelin or Nissl-stained histology. Note, this can also be observed when comparing 2D structure tensor and PLI data, suggesting it is a primarily a characteristic of the inputted microscopy data, rather than a feature of the hybrid method itself. This difference in crossing fibres is likely due to the nature of PLI which becomes less informative when a pixel (covering a volume of 4 × 4 × 50 μm with 50 μm being the tissue thickness) contains multiple fibre populations in which case PLI tends to output the orientation of the more dominant fibre population (Dohmen et al., 2015). In comparison, the histology data has a finer spatial resolution at 0.28 μm/pixel, where around 200 histology pixels fit into a single PLI pixel (4 μm/pixel), and so can likely better delineate crossing fibre populations (Rowe et al., 2013). The crossing fibre ratio may also be impacted by the size of the Gaussian kernel used in the structure tensor analysis, where in BigMac we used a kernel of *σ =*10 pixels ∼ 3 μm, the effect of which warrants further investigation. Future studies should focus on further validating the hybrid outputs across different microscopy contrasts, including comparisons with tracer connectivity data where our hybrid FODs provide new opportunities for validating the structure tensor parameters. Careful consideration should be taken when choosing the microscopy data for hybrid analysis as different microscopy contrasts can produce different results and provide specificity to different microstructural features. For example, the myelin-stain histology only includes myelinated structures, missing contributions from unmyelinated axons. Nissl-stain in the white matter, on the other hand, visualises glial cell bodies, where oligodendrocytes and astrocyte soma tend to be oriented in parallel to the surrounding axons (Baumann and Pham-Dinh, 2001; Johansen-Berg and Behrens, 2014). Future work into hybrid outputs from different microscopy contrasts may provide novel neuroanatomical insight into microstructure organisation.

The hybrid MRI-microscopy method has several limitations. First, it relies on the combined analysis of MRI and microscopy which should be carefully co-registered. We found that tracts reconstructed from the hybrid FODs in the most anterior part of the brain such as the uncinate fasciculus in Fig. 5 had less anterior reach compared to the dMRI reconstructions, which in this case more closely followed neuroanatomical expectations. This may be related to errors in the dMRI-microscopy co-registration in this region and/or errors in PLI orientations affected by background gelatine on the anterior slides, though this was primarily found to affect orientations in the grey matter rather than the more myelinated white matter where tissue birefringence tended to dominate (Supplementary figure 10). dMRI-microscopy co-registration in BigMac was achieved via a state-of-the-art registration tool called TIRL (Huszar et al., 2023a). However, due to the limited anatomical information at the most anterior or posterior brain (where sections show little or no white/grey matter contrast), the registration accuracy with these sections is lower. Second, the hybrid method does not provide ground truth information about the tract structure, thus requiring careful consideration when interpreting the neuroanatomical implications of its outputs. Other technology such as micro-CT can provide fibre orientations in both 2D and 3D at micrometer level, and so may provide validation opportunities for the hybrid tractography (Karamov et al., 2020; Masíset al., 2018). Third, our hybrid orientations will be affected by the spatial resolution of the dMRI. BAS model provides multiple orientations per voxel, capturing the range of fibre directions but not their spatial locations, and microscopy data “super-resolve” dMRI information. There is a trade-off during dMRI acquisitions between higher spatial resolution and higher angular resolution within a limited time. The former reduces partial volume effects, and the latter improves crossing fibre estimation and SNR. The optimal trade-off for hybrid tractography likely depends on the fibre tract, with high spatial resolution benefiting smaller tracts and high angular resolution aiding crossing fibre regions (Jones et al., 2020). Fourth, direct in vivo translation of the hybrid method presented here would be challenging, as it is difficult to obtain microscopy data in vivo. To circumvent these limitations, ongoing work in our lab focuses on using machine learning methods to translate the benefits of the hybrid method in vivo, when only limited dMRI is available.

There is ongoing work to further improve the hybrid MRI-microscopy method. First, in our current method, each hybrid orientation is considered independently from its surrounding neighbours. Here some similarity or neighbourhood constraint could be used to enforce local smoothness for very high-resolution reconstructions. Second, the BigMac data we used to reconstruct hybrid orientation has only semi-dense sampled microscopy, with a gap of 350 *μ*m between consecutive PLI slides. Therefore, our microscopy only partially explains the fibre orientations within a voxel and our ability to reconstruct FODs at high resolution is limited by the effective through-plane resolution (350 *μ*m gap). As BigMac includes both Nissl- and myelin-stained histology as well as PLI, future work can combine the three microscopy contrasts into a single hybrid FOD to further increase the through-plane resolution/sampling. Third, one benefit of our method is that we have both high spatial and angular resolution for the hybrid orientations. As such, we may benefit from fitting higher-order spherical harmonics to our hybrid FODs which may further improve our ability to capture small separation angles between multiple fibre populations. Fourth, our current method for hybrid orientation estimation did not consider the PLI retardance, which could inform the accuracy of the PLI orientation estimation. This could be considered in future implementations to down weight the microscopy orientation information in regions of higher uncertainty.

The hybrid outputs (FODs at different resolutions, provided as NIFTI files) are made openly available to the wider community and offer new opportunities for microscopy-informed, whole-brain tractography. The outputs can be utilised for novel neuroanatomical investigations, or to validate and drive in vivo MRI acquisition and analysis (e.g. by helping define the spatial resolution required to observe certain characteristics of the white matter). For example, future work could use the hybrid method to study the superficial white matter (Guevara et al., 2020) that connect neighbouring cortical regions with a U-shaped trajectory, as well as fibre tracking within subcortical regions (Maller et al., 2019). Though these regions represent challenging areas for diffusion tractography, the hybrid method based on high-resolution microscopy, coupled with the high specificity to axons, may provide superior fibre tracking.

The successful application of our method to different microscopy contrasts opens avenues for hybrid tractography to be performed on other multi-modal datasets that previously could not be used to perform 3D tract reconstruction (Budde and Annese, 2013; Mollink et al., 2017). This includes data acquired from other species facilitating cross-species investigations into structural connectivity, and small tissue blocks that focus on a single white matter bundle or region of interest (Schilling et al., 2019b; Tendler et al., 2022). For a microscopy dataset without dMRI, it is even possible to input dMRI data from another brain or atlas into the model, though the results may be less reliable due to the mismatch of the underlying brain microstructure. Crucially, our method relies on data that can and are being acquired in the human brain (eg. Big Brain project) (Amunts et al., 2013). Future whole-brain suitable human data will provide exciting opportunities for hybrid reconstruction of the microscopy-informed human connectome, and cross-species comparisons using the macaque data presented here.

## Data Availability

The pre-processed BigMac data with MRI and microscopy has been previously made openly available via the Digital Brain Bank (https://open.win.ox.ac.uk/DigitalBrainBank/#/). Whole-brain volumes of the fibre orientation distributions output from the hybrid method is made openly available on the Digital Brain Bank via a data sharing agreement to ensure the data is used for purpose which satisfy research ethics and funding requirements. This includes dMRI-PLI, dMRI-Gallyas, dMRI-Cresyl FODs at multiple spatial resolutions (1, 0.6 and 0.4 mm isotropic), and dMRI FODs at 0.6 mm. The code for constructing the hybrid orientation from dMRI and microscopy is accessible via (https://git.fmrib.ox.ac.uk/srq306/hybrid_microscopy_dmri). The code was written in MATLAB with further tutorial documentation and code explanation provided at (https://open.win.ox.ac.uk/pages/srq306/hybridtractographydoc/). The MATLAB scripts can be edited for other datasets with fairly minimal effort while future efforts aim to package it into a more widely applicable toolbox.
